# Mitigating the seductive details effect by topic and irrelevance signals

**DOI:** 10.1111/bjep.70022

**Published:** 2025-08-21

**Authors:** Lukas Wesenberg, Felix Krieglstein, Sebastian Jansen, Sascha Schneider, Günter Daniel Rey

**Affiliations:** ^1^ Psychology of Learning with Digital Media, Institute for Media Research Faculty of Humanities, Chemnitz University of Technology Chemnitz Germany; ^2^ Educational Technology, Institute of Education Faculty of Arts and Social Sciences, University of Zurich Zurich Switzerland

**Keywords:** cognitive theory of multimedia learning, irrelevance signals, seductive details, signalling, text comprehension, topic signals

## Abstract

**Background:**

Seductive details (interesting digressions in learning materials) are often integrated into learning units to make them more appealing to learners. However, studies indicate that this tends to overload students cognitively and impairs their learning performance.

**Aims:**

The present study investigated whether these negative consequences can be mitigated if seductive details are marked as thematically independent from the rest (topic signals) or as thematically independent and learning‐irrelevant (topic and irrelevance signals).

**Sample and Method:**

A total of 195 students read a text on the formation of coral reefs. Depending on the experimental condition, the text either included no seductive details (1), unsignalled seductive details (2), seductive details marked by topic signals, that is, placed in coloured boxes that were declared thematically independent (3) or seductive details marked by topic and irrelevance signals, that is, placed in coloured boxes that were declared thematically independent and irrelevant for the learning goal (4).

**Results:**

Results showed that topic signals mitigated and possibly even offset the detrimental effect of seductive details on transfer performance. Additional signalling of their irrelevance showed no significant effect. Furthermore, it was shown that including seductive details partially improves learning indirectly by increasing interest.

**Conclusions:**

The results suggest that the seductive detail effect cannot be explained solely by simple distraction. They provide a first indication that designers of (digital) textbooks who wish to increase students' interest by including digressions could prevent negative consequences by only signalling their thematic independence. However, this novel effect needs replication before reliable, practical recommendations can be made.

## INTRODUCTION

In order to make a lesson more interesting, teachers or instructional designers often incorporate entertaining digressions in learning materials that are only superficially related to the learning topic and, therefore, irrelevant to achieving the learning goal in terms of content. For example, teachers could include interesting anecdotes or fun facts about the uses and benefits of corals in a learning text that aims to explain the formation of coral reefs. Such digressions are also called seductive details. Studies have found evidence for the motivational benefits of seductive details. Amongst others, seductive details reduced negative affect and raised interest in, as well as satisfaction with the learning unit (e.g. Sitzmann & Johnson, 2014; Sung & Mayer, 2012; Wesenberg, Jansen, et al., 2025). In some cases, these affective improvements also mediated conducive effects on learning performance (Magner et al., 2014; Sitzmann & Johnson, 2014; Wesenberg, Jansen, et al., 2025). However, studies also show that including seductive details has certain disadvantages which seem to generally outweigh the motivational benefits with regard to learning performance as students presumably are cognitively overwhelmed by processing these additional materials (e.g. Brock et al., 2025; Garner et al., 1989; Harp & Mayer, 1998; Wesenberg, Krieglstein, et al., 2025). Hence, meta‐analyses showed that including seductive details mostly impairs learning performance (Rey, 2012; Sundararajan & Adesope, 2020).

**FIGURE 1:**
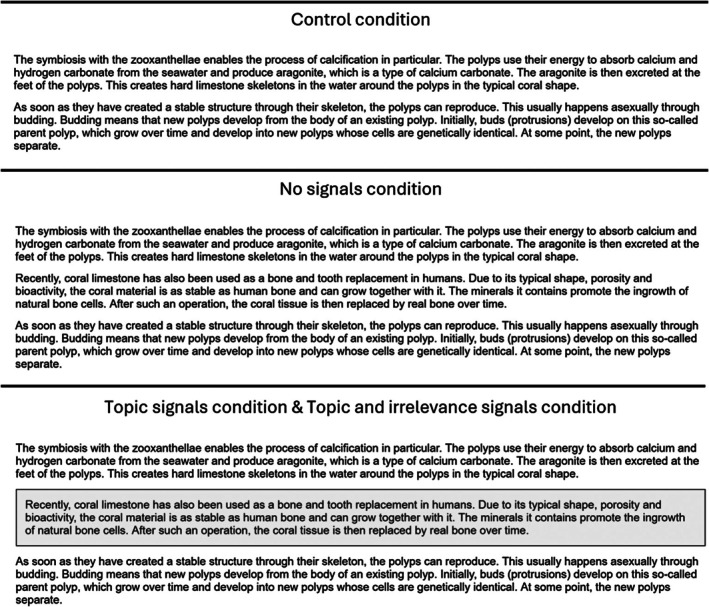
Learning page per condition (excerpts). Translated from German. The second paragraph in the no signals, the topic signals, as well as the topic and irrelevance signals condition represents a seductive detail.

Therefore, the present study was dedicated to the question of how to mitigate the negative cognitive effects without sacrificing the affective benefits of presenting seductive details. Specifically, it was examined if this can be achieved by two signalling methods: first, by topic signals, in which paragraphs with seductive details are placed in coloured text boxes and declared as thematically independent from the rest of the text by pre‐reading instructions; second, by topic *and* irrelevance signals, in which the same seductive detail paragraphs are also placed in coloured text boxes but declared as thematically independent *and* irrelevant to the present learning goal by those pre‐reading instructions. See Table 1 for definitions of topic and irrelevance signals, Table 2 for their exact implementation in the pre‐reading instructions, and Figure 1 for their exact implementation on the learning page.

**TABLE 1:** Definition of signalling methods and their general implementation in the present study.

Signalling method	Definition	Implementation on the learning page	Implementation in the pre‐reading instructions
Topic signals	Specific contents are marked as thematically independent from each other	Seductive details are placed in grey text boxes	Topic of grey text boxes and their thematical independence from the rest is declared
Irrelevance signals	Specific contents are marked as irrelevant	Seductive details are placed in grey text boxes	Grey text boxes are declared as irrelevant for learning goal and answering test questions

**TABLE 2:** Pre‐reading instructions per condition.

Condition		
Control & no signal	Topic signal	Topic and irrelevance signal
Read the text on the next page carefully. You will then have to answer test questions. The formation of coral reefs is explained.	Read the text on the next page carefully. You will then have to answer test questions. The formation of coral reefs is explained. Information on the benefits of corals is also included. These are marked with grey boxes, as they are independent of the rest in terms of content.	Read the text on the next page carefully. You will then have to answer test questions. The formation of coral reefs is explained. Information on the benefits of corals is also included. These are marked with grey boxes, as they are independent of the rest in terms of content. The information in the grey boxes is not relevant to the learning objective and is not asked in the test.

*Note*: Translated from German.

### Explaining the seductive details effect

The negative consequences of embedding seductive details in a learning unit can be explained by cognitive learning theories. In cognitive load theory (Sweller, 1994; Sweller et al., 1998), learning is understood as the construction and automation of schemata. Constructing schemata means that several information elements are structured into one superordinate unit of meaning. For example, when learning with a text about coral reef formation, the descriptions of the various individual steps that lead to the formation must be coordinated, subsumed and stored as the single schema ‘coral reef formation’. Such schemata are stored in long‐term memory and can be retrieved when needed. The construction of schemata takes place in working memory, which is limited in its capacity (Baddeley & Hitch, 1974). This means that only a limited number of elements can be processed simultaneously and related to each other in working memory. If learners are confronted with too many elements and the cognitive load is too high, the learning process is disturbed (Sweller et al., 1998). In this context, cognitive load theory differentiates between the cognitive load that is essential (intrinsic cognitive load) and that which is irrelevant (extraneous cognitive load) for understanding the learning topic (Kalyuga, 2011; Sweller et al., 2019). Harp and Mayer (1998) hypothesize that seductive details can increase extraneous processing in three different ways and thereby worsen learning performance.

First, learners may be distracted by seductive details when mentally turning towards the important material. In learner‐paced learning units, students possibly invest more time overall on the entire learning unit due to the seductive detail information that is additionally presented. However, in the case of distraction, learners might process the actual learning‐relevant parts less intensively or extensively due to the extra processing of seductive details. Accordingly, studies have shown that learners spend less time processing the relevant material when seductive details are presented, while some of them also proved that this mediates the detrimental influence of seductive details on learning performance (e.g. Korbach, 2016; Lehman et al., 2007; Sitzmann & Johnson, 2014).

Second, according to Harp and Mayer (1998), learners can be disrupted by seductive details when processing multiple information elements that relate to each other in some way. In this case, the embedding of seductive details throughout the learning material makes it difficult for learners to build a coherent mental model. For example, after reading about the first step and before reading about the second step in the coral reef formation, students may read interesting information about the use of coral in medicine. First, due to this interruption, they may experience more trouble integrating the second step in the right place within the entire causal chain, that is, they lose sight of which steps build upon another and which do not. Second, due to this interruption, they may experience more trouble categorizing terms in the second step section that were already previously explained in the first step section. Accordingly, two studies have shown that learners take longer to process learning‐relevant sentences presented immediately after seductive details than other relevant sentences, which could be an indicator of disruption processes taking place (Lehman et al., 2007; McCrudden & Corkill, 2010).

Third, according to Harp and Mayer (1998), learners can be diverted by seductive details when they are looking for superordinate theme(s) (i.e. a basic structure or a common thread) in the learning text to activate a useful schema that guides them in further text processing. Such schemata build on prior knowledge. For example, prior knowledge about the learning topic (e.g. corals) or prior knowledge about typical structures in learning text (e.g. how causal chains are represented). This kind of processing is also called top‐down processing (Kintsch & van Dijk, 1978; Neisser, 1967). If the superordinate theme is correctly recognized, the application of the respective guiding schema eases the text processing for learners in several ways: Learners can anticipate following information, infer helpful context‐information that is not directly verbalized in the text, know which information needs to be integrated with each other, and what should be retained and concluded from the text (sections) (Kintsch & van Dijk, 1978; McCrudden & Schraw, 2007; Sweller et al., 1998; Zwaan & Radvansky, 1998). In the case of diversion, due to the thematically divergent content of the seductive details, learners may either waste cognitive resources until they recognize that they better use different schemata for different sections or erroneously apply one inappropriate schema for processing the entire material. For example, one schema based on the core sections (e.g. formation of coral reefs) when processing seductive detail sections (e.g. uses of corals), one schema based on the seductive detail sections when processing core sections, or they use one rough and imprecise schema that subsumes the topics of the core and the seductive details sections (e.g. description of corals). All these scenarios interfere with the several advantages of activating an appropriate guiding schema that were listed above. A study by Garner et al. (1989) can be interpreted as support for this explanatory approach. In this study, students who did not receive seductive details named significantly more correct core idea units when asked to report ‘just the really important information […]’ (p. 47) after they read a text. In addition, a qualitative analysis showed that learners from the condition with seductive details often gave a mixture of core ideas and seductive detail ideas as an answer.

### Mitigating the seductive details effect by signalling

One way to prevent or mitigate the damage of seductive details on learning performance could be to use signalling methods. Signalling refers to the use of specific cues or markers to either highlight relevant elements in a learning material or to clarify the organization of that material (Meyer, 1975; Schneider et al., 2018; Van Dijk, 1979).

Relevance signalling is assumed to reduce cognitive load, as learners are guided in selecting information essential for understanding the learning topic and achieving the learning objective (Mautone & Mayer, 2001). Organizational signalling is assumed to reduce cognitive load by guiding learners in the organization of mental models (Harp & Mayer, 1998; Meyer, 1975). To this end, organizational signalling can be used to indicate logically separable information units. Additionally, it is possible to indicate how exactly the relations between the information units are to be understood, such as a causal chain, hierarchy, sequence or as not only separable but also independent information units (as for example in the case of topic signals). Studies have already investigated if different subforms of relevance or organizational signalling can moderate the effect of seductive details on learning:

First, studies examined the moderating effect of using typographical cues to signal the relevance of key content as opposed to other relevant or seductive detail content. However, neither the use of bold nor the use of italic font to signal the relevance of keywords significantly attenuated the seductive details effect (Abercrombie et al., 2019; Harp & Mayer, 1998).

Second, studies examined the moderating effect of using numbering cues to signal the organization of key content. However, the numbering of important sentences describing the steps of a causal chain in a learning text to signal that these parts build on each other did not significantly attenuate the seductive detail effect (Harp & Mayer, 1998).

Third, studies examined the moderating effect of using relevance instructions before the learning unit to signal the relevance of specific key content or generally relevant content (McCrudden & Schraw, 2007). The findings here were mixed. McCrudden (2019) confirmed that students recall more important core ideas of a text, including seductive details, when they are presented with some pre‐questions related to the text's main ideas and are asked to focus on these questions while reading. In line with this finding, Wang et al. (2024) showed that previewing test items reduced the negative effect of using decorative instead of representational pictures in learning units. However, Harp and Mayer (1998) could not confirm that stating learning objectives and briefly describing the test questions directly before the learning unit moderated the seductive detail effect. Tislar and Steelman (2021) even found that the detrimental effect of highly interesting SDs on test performance was significantly stronger when learners were provided with general learning objectives.

Fourth, studies examined the mitigating effect of using topic signals (see Table 1) to signal the thematical independence between all the relevant content and the irrelevant seductive detail content. The reason to use such topic signals is twofold. First, while seductive details might still *interrupt* learners, they should be less *disrupted* since they can quickly refer back to the section they read before the interruption if they trouble to integrate the new text section within the bigger picture or to categorize previously explained terms. Second, topic signals should also reduce diversion, as learners will not try to subsume all text sections through one guiding schema but know that they have to activate multiple independent schemata for the thematically different sections of the material. Kienitz et al. (2023) investigated the effect of separation prompts, which can be understood as topic signals. In this condition, learners were informed that the graphically marked materials on the formation and the unmarked materials on the consequences of lightning are thematically different and were asked to treat them separately. However, these prompts could not significantly reduce the detrimental seductive detail effect. This might potentially be explained by the fact that the seductive details examined were photographs and associated captions, which may have stood out slightly from the rest of the material due to the format alone and already suggested to learners to separate learning material and seductive details in terms of content. Hence, topic signals might be more useful when applied to pure seductive detail text embedded in typographically identical learning text as in the present study.

Lastly, studies examined the mitigating effect of using (ir)relevance signals (see Table 1) to signal the relevance of all the relevant content as opposed to the irrelevant seductive detail content. In contrast to relevance instructions, (ir)relevance signals are to be understood as those that specifically mark the (ir)relevant content instead of only providing cues/instructions beforehand. It is reasoned that if learners know which content within a learning environment is irrelevant to learning, they will probably ignore it as far as possible or at least invest less time and effort to process this content. Regarding the motivational benefits of seductive details, this might reduce the positive effect on situational interest compared to using only topic signals. However, regarding the cognitive disadvantages, this should not only further reduce the probability of disruption and diversion but also the probability of distraction and thereby foster learning even more as compared to using only topic signals. This kind of signalling mostly produced significant positive findings. In studies by Eitel et al. (2019) and Kienitz et al. (2023), a relevance signal condition was included that marked relevant text and illustrations by a red frame in contrast to the irrelevant seductive pictures and captions. Both studies confirmed that learning performance was impaired only in the seductive detail condition that did not receive these relevance signals. Subjects in the relevance signals condition also reported that they spent less time processing the seductive details, which explained the conducive signalling effects according to mediational analysis (Kienitz et al., 2023). Bender et al. (2021) replicated the effect on learning performance with similar instructions but a different learning topic and coloured text boxes marking the irrelevant material instead of red frames marking the relevant material.

### The present study

The present study aimed to investigate if the negative influence of seductive details can be mitigated by topic and irrelevance signals. While there is no empirical evidence for the usefulness of topic signals so far, former studies have already confirmed the benefit of (ir)relevance signals (Bender et al., 2021; Eitel et al., 2019; Kienitz et al., 2023). However, the investigated (ir)relevance signals conditions also contained either an explicit topic signal, as the pre‐reading instructions pointed out that the irrelevant content related to a different subtopic (consequences instead of the formation of lightning; Eitel et al., 2019; Kienitz et al., 2023) or at least an implicit topic signal (Bender et al., 2021) as the mere indication that the content is irrelevant to the learning objective presumably conveys to learners that the content is thematically different and should be understood independently of the rest. It can be assumed that irrelevance signals always imply at least a vague topic signal.

Hence, from the current research it is not yet clear whether topic signals, irrelevance signals, or only both together can attenuate the detrimental seductive detail effect. This is an important question, not least because it remains unsure if the effect can only be mitigated if learners are led to ignore the seductive details by highlighting them as irrelevant, which presumably also diminishes the motivational benefits of seductive details on learners' interest and affect, which is the reason to include them in the first place (e.g. Sitzmann & Johnson, 2014; Sung & Mayer, 2012; Wesenberg, Jansen et al., 2025).

Therefore, in the present study, we adapted an experimental design that includes four hierarchically ordered conditions to analyse the unique effect of using topic signals and the unique effect of using irrelevance signals in addition to topic signals. We used this hierarchical design instead of comparing a topic signal only and a relevance signal only condition to reduce the risk of confounding due to the assumed naturally implied topic signal in all irrelevance signal conditions (see explanation above).

1. *Control condition*:
  Learning text only
2. *No signals condition*:
  Learning text + seductive details
3. *Topic signals condition*:
  Learning text + seductive details + topic signals
4. *Topic and irrelevance signals condition*:
  Learning text + seductive details + topic signals + irrelevance signals.

Based on the literature and theoretical considerations discussed above, we propose several hypotheses that all only relate to comparisons between two consecutive factor levels due to the hierarchical nature of the independent variable.

#### Hypotheses regarding seductive details

Students from the control condition …

- … show better retention performances …
- … show better transfer performance …
- … show less extraneous cognitive load …
- … show less interest in the learning material …
- … invest less time on the entire learning page …

… than students from the no signals condition.

#### Hypotheses regarding topic signals

Students from the no signals condition …

- … show worse retention performances …
- … show worse transfer performances …
- … show more extraneous cognitive load …

… than students from the topic signals condition.

#### Hypotheses regarding irrelevance signals

Students from the topic signals condition …

- … show worse retention performances …
- … show worse transfer performances …
- … show more extraneous cognitive load …
- … show higher interest in the learning material …
- … invest more time on the entire learning page …
- … read a higher percentage of seductive details …

… than students from the topic and irrelevance signals condition.

#### Mediational hypotheses

Adding seductive details …

- … impairs test performance via increased extraneous cognitive load.
- … improves test performance via increased interest.

Adding topic signals …

- … improves test performance via decreased extraneous cognitive load.

Adding irrelevance signals …

- … improves test performance via decreased extraneous cognitive load.
- … impairs test performance via decreased interest.

## METHOD

### Design

The present study adopted a one‐factor design with four conditions. These differed regarding pre‐reading instructions and the content of the learning page. See Table 2 for a comparison of the pre‐reading instructions and Figure 1 for a comparison of the learning pages per condition.

The first condition, referred to as the *control condition* (*n* = 45), received standard pre‐reading instructions (see Table 2) comparable to those used in other studies examining the seductive detail effect and was presented with the learning text only on the learning page. The learning text concerned the formation of coral reefs. It comprised 461 words and was divided into eight paragraphs. The original as well as the English version of the text can be found here via OSF: OSF Link.

The second condition, referred to as the *no signals condition* (*n* = 49), received the same pre‐reading instructions as the control condition. However, regarding the learning page, in addition to the learning text, they were also presented with seductive details. The seductive details contained interesting information about the uses and benefits of corals that was irrelevant to reaching the learning goal as well as answering the test questions. Each of the five seductive details contained between 37 and 56 words, formed a separate paragraph and was inserted at points in the learning material to which it superficially related The original as well as the English version of the seductive details can be found here via OSF: OSF Link.

The third condition, referred to as the *topic signals condition* (*n* = 49), received the same pre‐reading instructions and seductive details as the no signals condition. However, the seductive details were marked by grey boxes (see Figure 1) and the pre‐reading instructions additionally explained the meaning of the grey boxes by specifying the topic of their content and emphasizing their independence from the rest (see Table 2).

The fourth condition, referred to as the *topic and irrelevance signals condition* (*n* = 52), received the same pre‐reading instructions and learning page as the topic signals condition. However, similarly to Eitel et al. (2019), the pre‐reading instructions additionally explained the irrelevance of the content in the grey boxes for the learning goal and learning test (see Table 2).

### Sample

Prior to the study, a power analysis using the G*Power software (Faul et al., 2007) for an analysis of covariance (ANCOVA) with four groups, an estimated medium effect size of *f* = .25, a power of 1 − *ß* = .8 and an alpha level of *α* = .05 resulted in a sample size of *N* = 179.

The sample was recruited by Prolific Academic Ltd., an online data collection platform that has been shown to generate high data quality (e.g. Peer et al., 2022). Five screening criteria were determined in the search for participants to ensure a homogeneous sample. These were current residence in Germany, German as fluent and primary language, current student status as well as a 100% approval rate for former study participations on prolific. Furthermore, participants needed to adhere to and confirm the conditions of participation, which means conducting the study in one piece without interruption in a quiet place via PC, laptop or tablet. Every participant was compensated with 2.70£/GBP.

A total of 215 students took part in the experiment. From this sample, 16 students had to be excluded since they reported at the end of the survey that they either took notes during the learning phase, which was not allowed, or they had used unauthorized aids to answer the test questions (e.g. Google, ChatGPT). One student was excluded as box‐plot analysis indicated him or her as an extreme outlier (Q3 + 3 * IQR) regarding the time taken for the learning and test phase of the experiment, suggesting that these phases were not completed without interruption. Additionally, three students had to be excluded because they failed at an attention check item implemented in the questionnaire. Therefore, the final sample consisted of 195 participants. The mean age was *M* = 24.95 (*SD* = 4.36); 39.5% identified as female, 57.4% as male and 3.1% as non‐binary. Regarding the educational programme, 46.2% of the students were enrolled in an undergraduate degree, 19.5% in a graduate degree, 3.6% in a doctoral degree, 23.6% attended a secondary school and the remaining 7.1% were enrolled in other programmes. Prior knowledge about the learning topic, that is, the formation of coral reefs, was very low, as indicated by a pretest (range from 0 to 13; *M* = .12; *SD* = .43). All participants were randomly assigned to one of the four conditions.

### Prestudy

When creating the seductive details, care was taken that they matched the definition of seductive details: That means they were (a) only tangentially related to the topic, (b) irrelevant for reaching the learning goal (i.e. answering the test questions) and (c) presumably interesting for learners. Accordingly, the content of all seductive details related to the use and benefits of corals. Interestingness was pretested before the main study through an online survey (*N* = 20). Like the primary study sample, Prolific recruited them using the same screening criteria. Five text segments representing the presumed seductive details were created for this pretest. In the survey, participants were presented with the presumed seductive details and a segment from the learning text that was similar in length (61 words). This segment was supposed to work as a reference item to validate the interestingness of the presumed seductive details. Survey participants were instructed to rate each text segment one after another for its interestingness on a seven‐point Likert scale from (1) *not at all interesting* to (7) *very interesting*. The presentation order of the text segments was rotated after each trial. One‐sided paired *t*‐tests confirmed that all of the presumed seductive details were significantly more interesting than the reference item (all *p*‐values < .036, all Cohen's *d* values >.43). Furthermore, one sample *t*‐test showed that the mean values of all presumed seductive details were rated significantly higher than the value of 4, which represented the centre of the scale (all *p*‐values < .021; all *M* > 4.59). Hence, the five presumed seductive details were confirmed to be interesting.

### Instruments

#### Prior knowledge

To measure prior knowledge, participants were asked to name all the known steps in the formation of coral reefs. The open‐ended answers were evaluated based on a rating sheet that listed all steps. For each correctly named step, participants were awarded one point. A total of 13 points could be reached. Two raters independently rated the answers of 25% of the sample in order to check the reliability of the rating sheet. There was almost perfect agreement between the two raters (κ = .85). Therefore, the rest of the answers were rated by one of the raters.

#### Working memory capacity

Working memory capacity was measured through an adapted version of the reading span task by Oberauer et al. (2000). Participants were presented with five short sentences that contained four to seven words and ended with a noun. Some of the sentences were semantically correct (e.g. ‘The flower smells wonderfully of spring’), and some were incorrect (e.g. ‘The fridge dances with the ocean.’). The sentences were presented sequentially for 3 seconds, followed by a one‐second transition period with a blank screen. Participants were given two assignments beforehand: First, they were instructed to decide during the three‐second presentation period of each sentence if it was semantically correct or incorrect by clicking on a yes or no button right below the sentence. This assignment was considered a distractor task. Second, participants were instructed to remember the last word of each sentence. This assignment was considered a primary task, that is, performance in this task is assumed to indicate the working memory capacity. After the fifth sentence, participants were asked to list all the words they remembered. Each participant's working memory capacity score was equivalent to the total number of correctly remembered words. Friedman and Miyake (2005) showed that this scoring method has better reliability and predictive power than other methods related to the scoring of reading span tasks. Internal consistency between correctly remembering each of the five words was rather low (*α* = .41) which may be attributed to the nature of construct measured, as persons with medium working memory capacity only can mentally hold on to some of the words at the expense of losing other words.

### Test performance

The learning test comprised measures of retention and transfer. The retention score was formed from a free recall, a cued recall and a recognition task (*α* = .83). In the free recall task, students were asked to name all the processes and stages in the formation of coral reefs that they could remember. A total of 13 points could be reached. In the cued recall task, students were asked to briefly describe what happens in the six processes explained in the learning text (settlement, metamorphosis, symbiosis with zooxanthellae, calcification, budding, spawning). For each process, three points could be achieved. In the recognition task, students were asked to put the different stages and processes in the correct hierarchical order, starting after the stage of larvae swimming in the sea. They received one point for correctly recognizing the first step and then, to avoid repetition errors, one point whenever two consecutive steps were correctly assembled one after another. A total of 10 points could be reached for this task. Accordingly, the maximum retention score to be achieved was 31. The transfer score was formed from six open‐ended questions (*α* = .65). These questions required students to draw inferences from the information provided in the learning text (e.g. ‘Most corals settle in so‐called euphotic, i.e. light‐flooded zones in waters. Why is this important for their energy production?’). Students were awarded one point for every correctly answered question. A total of six points could be achieved. All transfer questions can be found here via OSF: OSF Link.

For all three open‐ended (sub)tests, two raters independently rated the answers of 25% of the sample based on a rating sheet containing coding instructions in order to check on the reliability of this instrument. There was substantial agreement between the two raters regarding the free recall (κ = .70) and the cued recall task (κ = .67) as well as almost perfect agreement regarding the transfer task (κ = .88). Therefore, the rest of the answers were rated by one of the raters.

#### Extraneous cognitive load

Extraneous cognitive load was measured through the scale by Krieglstein et al. (2023) which demonstrated high validity and reliability. The scale consists of five statements on the learning unit which need to be evaluated on a 7‐point Likert scale ranging from (1) *not at all true* to (7) *completely true* (*α* = .92). Since learners are often unaware or misled about the actual theme or learning goal of a lesson which contains seductive details (Harp & Mayer, 1998), we slightly adapted the five statements in that we indicated the specific learning objective (e.g. ‘The design of the learning material made it difficult to quickly find important information on the formation of coral reefs’).

#### Interest

Schiefele's (1990) topic interest scale was adapted to measure interest in the learning material. The scale consists of eight items, that is, eight adjectives, with four targeting feeling‐related (interested, bored, stimulated, indifferent) and four targeting value‐related (meaningful, unimportant, useful, worthless) aspects of interest, which must be rated on a 7‐point Likert scale from *not at all* to *completely* concerning their suitability for the text provided on the learning page (*α* = .89).

#### Time invested on learning page

To measure the invested reading time (in seconds), the time that students spent on the learning page was tracked by the online platform Soscisurvey.

#### Percentage of seductive details read

To measure how much of the seductive detail content was read by the students in the topic signals condition and the topic and irrelevance signals condition, they were asked: ‘Approximately what percentage of the text in the grey boxes did you read through? Please answer honestly—the answers are anonymous and have no consequences for you or your compensation!’. Students answered the question through a slider with increments of 10. Students from the other two learning conditions were not asked this question since they either did not receive seductive details or could not know which content was supposed to be an seductive detail and which was not.

### Procedure

As *Prolific* was used for recruiting, potential participants who fit the screening criteria were informed via email that they could participate in this study. They accessed the learning environment via a link. *Soscisurvey* provided the learning environment. After they had been informed about the general procedure of the study and the conditions regarding participation, voluntariness, data security and use of the anonymized data, they were asked to give their consent to participate in the study and to switch on the full screen mode. Participants started with the reading span task. Next, they were asked for their prior knowledge. After that, students received the pre‐reading instructions and the learning page according to their conditions. In the following, measures of interest and extraneous cognitive load were collected before students worked on the retention tasks (free recall—cued recall—recognition), and lastly on the transfer task. Students from the topic signals condition as well as students from the topic and irrelevance signals condition were then asked for the reading time of the grey boxes (seductive details). The study closed with a questionnaire collecting demographic data. The mean time to complete the experiment was *M* = 18.11 min (*SD* = 7.62). At the beginning of the study, all participants were informed about the conditions of participation, which were based on APA ethical standards as well as the guidelines of the German Research Foundation (DFG) and the German Psychological Society (DGPs). This briefing included information about voluntariness, the right to withdraw their consent at any point, data protection and the anonymous analysis and use of their data. All students gave their informed consent. As the study was non‐medical, low‐risk research, no explicit approval was required from the responsible ethics committee at the Institute for Media Research at the Chemnitz University of Technology.

## RESULTS

The data were analysed using JASP (v0.18.3) and the SPSS version of PROCESS (v4.1). ANCOVAs with planned contrast and mediated regression analyses (Model 4, 95% CI, 5000 bootstrap samples) were conducted to test the hypotheses and research questions. The experimental condition was set as the independent variable in every analysis, and the pretest score and working memory capacity as covariates.

In line with the proposed hypotheses, the independent variable was recoded by a repeated (sequential) coding system, resulting in three contrast effects: The first contrast represents the effect of adding seductive details, thereby comparing the control and the no signals condition (1, −1, 0, 0). The second contrast represents the effect of adding topic signals, thereby comparing the no signals and the topic signals condition (0, 1, −1, 0). The third contrast represents the effect of adding irrelevance signals, thereby comparing the topic signals and the topic and irrelevance signals condition (0, 0, 1, −1). No outliers (Q1–3 * IQR or Q3 + 3 * IQR) were found for any dependent variables. Marginal means for all four conditions are reported in Table 3. Zero‐order correlations between all measures can be found in the Appendix (Table A1).

**TABLE 3:** Marginal means per condition.

	Experimental condition			
	Control (*n* = 45)	No signals (*n* = 49)	Topic signals (*n* = 49)	Topic and irrelevance signals (*n* = 52)
Retention	14.99 (.97)	12.81 (.93)	14.95 (.93)	13.90 (.90)
Transfer	2.90 (.22)	*1.82 (.21)	*2.96 (.21)	2.40 (.20)
Extraneous load	18.20 (1.18)	18.06 (1.14)	19.55 (1.14)	20.14 (1.11)
Interest	37.86 (1.27)	*42.49 (1.22)	41.67 (1.22)	41.72 (1.19)
Time on learning page	159.61 (20.58)	*250.89 (19.73)	234.69 (19.74)	222.66 (19.20)
Pct. of seductive details read	–	–	85.56 (4.63)	*68.99 (4.50)

*Note*: Standard errors are in parentheses. Means are adjusted for covariates (working memory capacity and prior knowledge). The mean values marked with an asterisk differ significantly (*p* < .05) from the mean values of the condition to their left. Due to the hierarchical design and sequential contrast system, only consecutive factor levels were compared.

### ANCOVA analyses

Regarding retention performance, the ANCOVA suggested no significant differences between the groups; *F*(3, 189) = 1.23, *p* = .301, $\eta_{p}^{2}$ = .02. Therefore, H1a, H2a and H3a were not supported.

Regarding transfer performance, the ANCOVA suggested significant differences between the groups, *F*(3, 189) = 6.42, *p* < .001, $\eta_{p}^{2}$ = .09. Consistent with H1b, planned contrasts showed that transfer performance was impaired in the no signals condition compared to the control condition, *p* < .001, *d* = .74. In turn, consistent with H2b, the topic signals condition improved transfer performance compared to the no signals condition, *p* < .001, *d* = −.79. For exploratory reasons, we were interested if the addition of topic signals only mitigated the detrimental seductive detail effect or even offset it. Hence, we employed Bayesian ANCOVA analyses in JASP to test the null hypothesis that the control and the topic signals condition do not differ. Balanced, default priors were used for both models (P(M) = .5). Results showed moderate evidence favouring the null over the alternative hypothesis, BF_01_ = 4.57 (Lee & Wagenmakers, 2013), suggesting that it is 4.57 times likelier that there are no differences between groups and, thus, that the topic signals offset rather than only mitigated the detrimental seductive detail effect. Contrary to H3b, there was no significant difference between the topic signals and the topic and irrelevance signals condition, *p* = .055, *d* = .39.

Regarding extraneous cognitive load, the ANCOVA suggested no significant differences between the groups; *F*(3, 189) = .82, *p* = .487, $\eta_{p}^{2}$ = .01. Therefore, H1c, H2c and H3c were not supported.

Regarding interest, the ANCOVA suggested significant differences between the groups, *F*(3, 189) = 2.76, *p* = .044, $\eta_{p}^{2}$ = .04. Consistent with H1d, planned contrast showed that students from the no signals condition showed more interest in the learning material than students from the control condition, *p* = .009, *d* = −.54. The topic signals condition did not significantly differ from the no signals condition, *p* = .636, *d* = .10. Contrary to H3d, students from the topic and irrelevance signals condition did not show significantly less interest than students from the topic signals condition, *p* = .976, *d* = −.01.

Regarding time invested, the ANCOVA suggested significant differences between the groups, *F*(3, 189) = 3.89, *p* = .010, $\eta_{p}^{2}$ = .06. Consistent with H1e, planned contrasts showed that students from the no signals condition spent more time on the learning page than students from the control condition, *p* = .002, *d* = −.66. The topic signals condition did not significantly differ from the no signals condition, *p* = .562, *d* = .12. Contrary to H3e, students from the topic and irrelevance signals conditions did not spend significantly less time on the learning page than students from the topic signals condition, *p* = .663, *d* = .09.

Regarding the percentage of seductive details read, consistent with H3f, the ANCOVA indicated that students from the topic signals condition read significantly more of the seductive details than students from the topic and irrelevance signals condition; *F*(1, 97) = 6.54, *p* = .012, $\eta_{p}^{2}$ = .06.

### Mediational analyses

**FIGURE 2:**
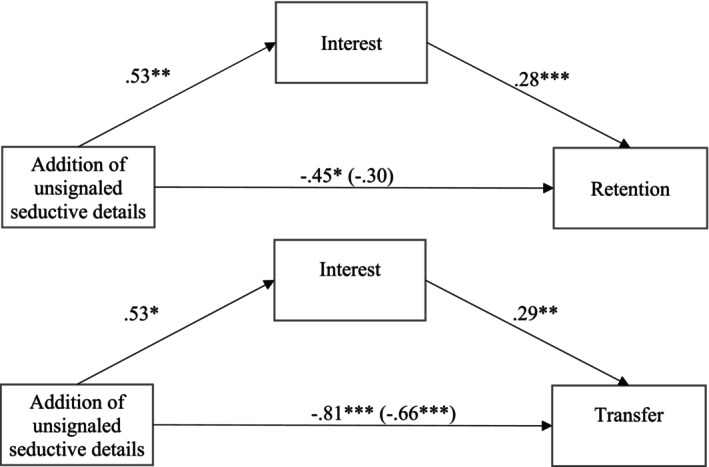
Diagrams for significant mediations. Numbers represent standardized regression coefficients. The coefficient for the total effect of the factor on retention is in parentheses. Prior knowledge and working memory capacity were included as covariates in this model. *p < .05, **p < .01, ***p < .001.

Regarding the addition of seductive details, contrary to H4a, the mediation analysis showed no significant partially standardized relative indirect effect via extraneous cognitive load on retention, *β* = .01; *SE* = .06; 95% CI [−.11, .12] or on transfer, *β* = .00; *SE* = .06; 95% CI [−.11, .12]. However, consistent with H4b, the mediation analysis showed a significant partially standardized relative indirect effect via interest on retention, *β* = .15; *SE* = .06; 95% CI [.04, .29], as well as on transfer, *β* = .15; *SE* = .07; 95% CI [.04, .30]. More specifically, adding seductive details resulted in higher interest, *β* = .53; *p* = .009. In turn, interest was associated with higher scores in retention performance, *β* = .28; *p* < .001, and higher scores in transfer performance, *β* = .29; *p* < .001. Mediation diagrams for these significant indirect paths can be found in Figure 2.

Regarding the addition of topic signals, contrary to H5a, the mediation analysis showed no significant partially standardized relative indirect effect via extraneous cognitive load on retention, *β* = −.05; *SE* = .06; 95% CI [−.19, .06] or on transfer, *β* = −.05; *SE* = .06; 95% CI [−.17, .06]. There was also no significant partially standardized relative indirect effect via interest on retention, *β* = −.03; *SE* = .06; 95% CI [−.14, .09] or on transfer, *β* = −.03; *SE* = .06; 95% CI [−.15, .09].

Regarding the addition of irrelevance signals, contrary to H6a, the mediation analysis showed no significant partially standardized relative indirect effect via extraneous cognitive load on retention, *β* = −.02; *SE* = .06; 95% CI [−.19, .06] or on transfer, *β* = −.02; *SE* = .05; 95% CI [−.13, .09]. Contrary to H6b, there was also no significant partially standardized relative indirect effect via interest on retention, *β* = .00; *SE* = .05; 95% CI [−.11, .10] or on transfer, *β* = .00; *SE* = .06; 95% CI [−.11, .11].

## DISCUSSION

This study aimed to investigate if the use of topic and irrelevance signals can mitigate the negative influence of seductive details on learning performance. In the following, the results on this question are summarized and interpreted; implications deduced, as well as aspects that limit their generalizability discussed.

### Interpretation

#### Results on test performance

Contrary to expectations, no significant effects were found regarding recall performance. However, confirming the classic seductive detail hypothesis, students who received seductive details performed worse on the transfer test than students who did not receive seductive details (e.g. Garner et al., 1989; Rey, 2012; Sundararajan & Adesope, 2020).

Furthermore, as hypothesized, this detrimental seductive detail effect was mitigated and potentially even offset when the seductive details were marked as thematically independent from the rest of the learning text by topic signals. It is reasoned that these topic signals were helpful to learners as they prevent detrimental diversion and disruption processes caused by the seductive details (Harp & Mayer, 1998). When learners know which specific parts of a learning text are thematically distinct, they presumably activate different guiding schemata for each of those parts to make sense of them instead of being diverted. Furthermore, if they get interrupted by seductive details, they can more quickly find and refer back to the last paragraph that the following paragraph thematically relates to, which helps to infer connections between those parts of the learning material.

However, contrary to expectations, marking the seductive details as instructionally irrelevant by irrelevance signals did not further improve transfer performance when seductive details were already marked by topic signals. This finding is in contrast to the theoretical considerations and the results from other studies (Bender et al., 2021; Eitel et al., 2019; Kienitz et al., 2023). However, the present null effect may also be attributed to the strong effectiveness of the topic signals, which already might have offset the detrimental seductive detail effect. Hence, it could be reasoned that irrelevance signals had no possibility to further improve learning in addition to topic signals. Therefore, the unique addition of irrelevance signals could still prove useful in a learning context, where topic signals alone are not enough to offset the seductive detail effect.

Besides that, the observation that adding irrelevance signals almost significantly even decreased transfer performance compared to the topic signals condition could be explained by sample characteristics. Differently from previous studies (Bender et al., 2021; Eitel et al., 2019; Kienitz et al., 2023), the subjects were recruited by Prolific and, therefore, possibly quite familiar with experimental studies, concerned with possible attention checks and may have been sceptical about the statement that the information in the grey text boxes is actually irrelevant. This could have led to uncertainty, task‐irrelevant thinking and poorer learning performance than expected.

#### Results on interest and cognitive load

As expected, adding seductive details raised interest in the learning material, which also mediated improvements in recall and transfer performance. These analyses confirm the findings of previous studies (Magner et al., 2014; Sitzmann & Johnson, 2014; Wesenberg, Jansen, et al., 2025) while they also contrast with others (e.g. Colliot & Boucheix, 2023) which might suggest that the choice of the content to be presented as seductive details is quite important if the aim is to trigger affective‐motivational processes. Nonetheless, the total effect on learning performance was negative, which indicates that there must be other, parallel mediating variables. Surprisingly, the perceived extraneous cognitive load could not be confirmed as such. This could be understood as a sign that it is not cognitive overload that drives the seductive detail effect. However, another reason may be the circumstance that extraneous load was collected via self‐report, just like in other studies which did not find significant effects either (e.g. Bender et al., 2021; Colliot & Boucheix, 2023). This can be particularly problematic in the context of seductive details, as learners would have to be aware of the learning objective and the exact content of the subsequent learning test to correctly classify seductive details as extraneous content, which is often not the case according to diversion theory (Harp & Mayer, 1998). Even though we specified the learning goal in formulating the items of the extraneous cognitive load scale, learners were still unaware of it during the learning phase, making accurate retrospective judgements difficult. Future studies might use less complex overall load instead of single load scales, which ask about specific definitions of cognitive processes that learners are not familiar with and that presumably overwhelm them.

#### Results on time variables

As expected, including seductive details in a learning text significantly raised the time learners invested on the learning page. Furthermore, in line with expectations, students reported reading less of the seductive detail passages when they were marked as learning‐irrelevant by irrelevance signals. This is in line with the results of Kienitz et al. (2023). However, similar to the Kienitz et al. study, the irrelevance signals did not result in the seductive details being wholly ignored. The average reported proportion of seductive details read was still relatively high in this group at just under 70%. This suggests that seductive details, possibly due to their high level of interest, can still hold students' attention to a certain extent, even if they are declared completely irrelevant.

However, the decline due to the irrelevance signals, as indicated by the specific self‐report measure, was not reflected by significant differences in the global objective measure on the total time spent on the learning page. This rather suggests that learners do not ignore irrelevance signalled seductive details more often (e.g. because of interestingness, curiosity or senses of duty) or even that learners were sceptical about the instructions in the present study, as explained above. However, the insignificance could also be explained by the fact that measures of time spent in timely unrestricted learning environments are easily subject to high standard deviations and standard errors, as in the present study (see Table 3). Taken together, the present study provides only inconclusive evidence on the question of how intensively and extensively learners process interesting digression when they are marked as irrelevant. Future research investigating the effect of irrelevance signals could use eye‐tracking, which provides a more accurate measurement and may have a lower risk of bias than the self‐report measure that was used in the present study.

### Implications

The findings of the present study confirm that seductive details have certain affective‐motivational benefits even though they nevertheless impair learning performance. However, this study also confirms that signalling the distinctiveness of seductive details by graphical markers mitigates these impairments (Bender et al., 2021; Eitel et al., 2019; Kienitz et al., 2023). In former studies, the signalled distinctiveness of seductive details (implicitly) related to thematical differences *and* their irrelevance for the learning goal. Adding to this state of evidence, the present study suggests that the seductive detail effect might already be mitigated by signalling *just* the thematical difference between the seductive details and the rest of the material without additionally signalling their irrelevance.

From a theoretical perspective, these results indicate that the seductive detail effect cannot be explained by distraction effects alone (Harp & Mayer, 1998). If it was only distraction, the influence of the seductive details in the topic signals condition would likely be the same as in the unsignalled seductive detail condition since the seductive detail information was only marked as thematically deviant but not as irrelevant to learning. Hence, the topic signalled seductive details can presumably be considered just as distracting as the unsignalled seductive details. Nonetheless, the topic signals successfully mitigated or even potentially offset the seductive detail effect, which speaks for the validity of the disruption and/or diversion hypothesis. Furthermore, the results regarding the mediational analyses provide support for the theoretical assumption that seductive details as digressions in learning text can elevate the interest in the entire learning unit and by that also bear motivational benefits regarding the learning performance of students besides cognitive detriments.

From a practical perspective, the results reveal first indications that teachers or instructional designers who want to arouse learners' interest with the help of seductive details could avert potential negative consequences on learning performance using topic signals, thereby marking their thematic independence. Hence, there might be no need to actively cue them to ignore such digressions. In this way, the positive affective benefits of seductive details (which were confirmed in this study) could be utilized while avoiding the adverse cognitive effects. Particularly, the results of this study might be applicable to the design of digital learning texts or textbooks. In this context, for example, seductive detail text could be placed in coloured text boxes to illustrate the independence of the content graphically.

However, as explained above, more research is needed before these implications can be used as a basis for making reliable practical recommendations. On the one hand, this is the first study to find significant beneficial effects of topic signals; replication is warranted. On the other hand, it remains unsure how these results translate to other learning contexts since this effect was found under specific conditions that limit its generalization. We discuss these limitations in the following and, thereby, outline directions for future research.

## LIMITATIONS AND FUTURE DIRECTIONS

First, the results of this study are limited in terms of generalization to other implementations of topic signals. For example, future studies could investigate whether a leaner signalling method is sufficient to achieve the desired effect. In the present study, we decided to combine graphical signalling using coloured text boxes with pre‐reading instructions explaining the general topic and the independent character of those boxes because we reckoned that any effective signalling requires that learners are aware of its meaning. However, for example, it could be that a graphical highlighting of the seductive details is enough, and no accompanying pre‐reading instruction is needed. Similarly, only a vague pre‐reading instruction that informs about the presence of thematically divergent content without graphically highlighting it could also be enough for learners to recognize and classify the corresponding text segments and mitigate seductive detail effects.

Second, it remains unsure if the beneficial topic signals effect can be generalized to other learning environments or other types of seductive details. For example, with seductive details that are only reproduced orally by teachers, and no graphical signalling is possible, or in the case of decorative images, where topic signals may be less critical because the distinctiveness of the seductive details from the rest of the material is more evident to learners anyway. Additionally, it would be interesting to see if topic signals also work in live lectures where learners have no possibility to refer back to relevant information elements that were presented before the seductive detail interruption. Another specific characteristic of this learning environment was that a prior knowledge test was administered before the learning phase, which may have acted as a productive failure prompt by drawing attention to knowledge gaps and may have led to a type of processing that would not be found in many real classroom contexts (Kapur, 2008).

Third, future studies could examine aptitude‐treatment interactions with regard to the signalling of seductive details. In particular, the effect of irrelevance signals might depend on several learner characteristics. For example, determined or ambitious learners might especially react with consequent ignorance towards the seductive details when they are informed about their irrelevance. Alternatively, the effect could be less pronounced in learners with a need for cognition, who feel the need to read and understand these sections despite the irrelevant information. Such learner characteristics were not observed and assessed in the present study, which could also be a reason for the non‐significant irrelevance signal effect.

Lastly, it remains unsure how the found effects generalize across different student samples. In the present study, a general sample of students, mainly consisting of university students, was invited. The study did not assess participants' specific field of study, ethnicity or socio‐economic background. Future studies might focus on more specific sample clusters or include such variables to provide a more nuanced picture about the generalizability, allow exploration of possible aptitude‐treatment interactions, and especially to control for other possible relevant a‐priori differences besides domain‐specific prior knowledge and working memory capacity, as in the present study.

## AUTHOR CONTRIBUTIONS

**Lukas Wesenberg:** Conceptualization; investigation; methodology; visualization; writing – original draft; formal analysis. **Felix Krieglstein:** Writing – review and editing. **Sebastian Jansen:** Writing – review and editing. **Sascha Schneider:** Writing – review and editing. **Günter Daniel Rey:** Writing – review and editing; supervision.

## CONFLICT OF INTEREST STATEMENT

The authors have no competing interests to declare that are relevant to the content of this article.

## Data Availability

The data that support the findings of this study are available from the corresponding author upon reasonable request.

## Appendix

**TABLE A1:** Zero‐order correlations between measures.

Variable	1	2	3	4	5	6	7
1. Working memory capacity							
2. Prior knowledge	.05						
3. Time invested	−.05	.12					
4. ECL	−.16*	−.05	−.22**				
5. Interest	.07	.17*	.29**	−.34**			
6. Retention	.40**	.25**	.18*	−.34**	.31**		
7. Transfer	.36**	.17*	.19**	−.31**	.28**	.73**	
8. Pct. of seductive details read	.08	.14	.33**	−.39**	.39**	.34**	.41**

* *p* < .05;

** *p* < .01.
